# Identification and Functional Analysis of Variant Haplotypes in the 5′-Flanking Region of Protein Phosphatase 2A-Bδ Gene

**DOI:** 10.1371/journal.pone.0035524

**Published:** 2012-04-23

**Authors:** Hui-Feng Chen, Li-Na Lin, Yu-Xi Chen, Jian-Xin Wan, Jie Luo, Chen-Zi Zhang, Xiao-Jie Li, Yao-Ming Hu, Jian-Rong Mai, Wen Chen, Zhong-Ning Lin, Yu-Chun Lin

**Affiliations:** 1 Faculty of Preventive Medicine, Guangdong Provincial Key Laboratory of Food, Nutrition and Health, School of Public Health, Sun Yat-sen University, Guangzhou, People's Republic of China; 2 Department of Medical Inspection, Sun Yat-sen University, The First Affiliated Hospital, Guangzhou, People's Republic of China; Virginia Commonwealth University, United States of America

## Abstract

Serine-threonine protein phosphatase 2A (PP2A) is a trimeric holoenzyme that plays an integral role in the regulation of cell growth, differentiation, and apoptosis. The substrate specificity and (sub)cellular localization of the PP2A holoenzymes are highly regulated by interaction with a family of regulatory B subunits (PP2A-Bs). The regulatory subunit PP2A-B/PR55δ (PP2A-Bδ) is involving in the dephosphorylation of PP2A substrates and is crucial for controlling entry into and exit from mitosis. The molecular mechanisms involved in the regulation of expression of PP2A-Bδ gene (*PPP2R2D*) remain largely unknown. To explore genetic variations in the 5′-flanking region of *PPP2R2D* gene as well as their frequent haplotypes in the Han Chinese population and determine whether such variations have an impact on transcriptional activity, DNA samples were collected from 70 healthy Chinese donors and sequenced for identifying genetic variants in the 5′-flanking region of *PPP2R2D*. Four genetic variants were identified in the 1836 bp 5′-flanking region of *PPP2R2D*. Linkage disequilibrium (LD) patterns and haplotype profiles were constructed for the genetic variants. Using serially truncated human *PPP2R2D* promoter luciferase constructs, we found that a 601 bp (−540 nt to +61 nt) fragment constitutes the core promoter region. The subcloning of individual 5′-flanking fragment revealed the existence of three haplotypes in the distal promoter of *PPP2R2D*. The luciferase reporter assay showed that different haplotypes exhibited distinct promoter activities. The EMSA revealed that the −462 G>A variant influences DNA-protein interactions involving the nuclear factor 1 (NF1). *In vitro* reporter gene assay indicated that cotransfection of NF1/B expression plasmid could positively regulate the activity of *PPP2R2D* proximal promoter. Introduction of exogenous NF1/B expression plasmid further confirmed that the NF1 involves in the regulation of *PPP2R2D* gene expression. Our findings suggest that functional genetic variants and their haplotypes in the 5′-flanking region of *PPP2R2D* are critical for transcriptional regulation of PP2A-Bδ.

## Introduction

Protein phosphatase 2A (PP2A) is a highly conserved serine/threonine phosphatase which may account for up to 1% of cellular protein and the majority of serine/threonine phosphatase activity in the cell. The physiological functions of PP2A have been implicated in all aspects of cellular processes [1], [2]. The PP2A heterotrimeric holoenzyme is composed of a catalytic subunit (PP2A-C), a scaffold subunit (PP2A-A) and one member of four families of regulatory subunits (PP2A-Bs). The structural (A) and catalytic (C) subunits form the constitutive core enzyme, which associates with one of a large number of regulatory B subunits. The PP2A-Bs subunits are derived from four diverse gene families (B, B′, B″, and B″′). There is little sequence similarity between families, but members of a family maintain high similarity. Given the large number of PP2A subunits, it is thought that each cell expresses a dozen or more distinct holoenzyme complexes, which act on a diverse array of substrates [1], [3].

The B family (B55, PR55) of regulatory subunits is encoded by four genes (*PPP2R2s*): Bα, Bβ [4], Bγ [5], [6], and Bδ [7]. B family members (PR55s) exhibit temporal and spatial expression patterns; B55α (PR55α) and B55δ (PR55δ) are expressed almost ubiquitously, while B55β (PR55β) and B55γ (PR55γ) are highly enriched in the brain [4], [5], [7]. The primary structures and functions of the B subunits are conserved from yeast to mammals [8]. The structures of the PR55 subunits are predicted to form β-propellers with divergent N-terminal tails that act as signals for subcellular targeting [9], [10], [11]. A structural feature of the PR55 subunits is the presence of five degenerate WD-40 repeats. WD-40 repeats are minimally conserved sequences of approximately 40 amino acids that typically end in tryptophan-aspartate (WD) and are thought to mediate protein-protein interactions [12].

*In vitro* dephosphorylation assays support a role for Bδ in the targeting of the PP2A heterotrimer to the dephosphorylation and inactivation of ERKs [13]. Mochida et al. reported that a particular form of PP2A containing a B55δ subunit (PP2A-B55δ) has antimitotic activity and is the major protein phosphatase to act on model cyclin-dependent kinase (CDK) substrates in *Xenopus* egg extracts [14]. An interplay between the *Drosophila* serine/threonine kinase Greatwall (gwl) and PP2A/Bδ during mitotic entry was recently observed in two separate studies [15], [16]. PP2A/Bδ activity prevents mitotic entry by maintaining Cdc25 in a dephosphorylated and inactive state [17]. Similar cell cycle regulatory activity has been observed with the mammalian ortholog of gwl, microtubule-associated serine/threonine kinase-like (MASTL); however, the human MASTL-PP2A interaction has not been characterized [17]. Together, these data indicate that PP2A/Bδ plays essential roles in the regulation of substrate dephosphorylation and in cell cycle signaling pathways.

Currently, no functional variants of the PP2A-Bδ subunit gene (*PPP2R2D*) have been found, and little is known about the regulation of PP2A-Bδ expression. Moreover, the allele distribution in specific population is yet to be defined and a functional characterization of genetic variants or single nucleotide polymorphisms (SNPs) in the 5′-flanking region of the *PPP2R2D* gene has not yet been undertaken. In this study, we identify four variants and multiple various haplotypes in the 5′-flanking region of *PPP2R2D* in the southern Han Chinese population and demonstrate that certain functional variants in diverse haplotypes and nuclear factor 1 (NF1) may regulate the transcription of *PPP2R2D* gene and the expression of PP2A-Bδ.

## Materials and Methods

### Study subjects

Seventy healthy Han Chinese donors were recruited for this study. The individuals were randomly selected from residents of Guangzhou city (Guangdong province, China) in southern China. At recruitment, each participant was personally interviewed to obtain detailed information on their demographic characteristic such as gender, age and related family background. All subjects were unrelated ethnic Han Chinese confirmed by ID cards and “Household Register books”, which serve as identification in China as described in our previous study [18]. After physical examination, 5 ml of blood was collected, labeled, and delivered to the laboratory for immediate DNA isolation. This study was approved by the ethics committee of the School of Public Health, Sun Yat-sen University, and written informed consent was obtained from each subject.

### Variants screening

Genomic DNA samples derived from peripheral blood were used to search for genetic variants within the 5′-flanking region (−1775 nt to +61 nt) of the *PPP2R2D* gene (the first nucleotide of the RNA transcript is defined as +1 nt). The 1836 bp 5′-flanking fragment of *PPP2R2D* gene from each subject was amplified by PCR using a pair of primers (Table 1). The PCR cycling conditions were 94°C for 5 minutes, followed by 35 cycles at 94°C for 30 seconds, 55°C for 30 seconds, and 72°C for 2 minutes, with a final extension step at 72°C for 10 minutes. Genetic variants were detected by direct sequencing of the purified PCR products with the ABI PRISM DYE Terminator Sequencing Kit (Applied Biosystems, USA) using three sequencing primers (Table 1) to sequence the complete fragment. Finally, the Sequencer program was used to identify genetic variants that were then confirmed by 2 independent observers. We further confirmed these variant positions and individual genotypes by reamplifying and resequencing the variant sites from the opposite strand.

**Table 1:** Primers used for cloning and sequencing of *PPP2R2D* gene promoter and *NFI/B* gene.

Primer name	Oligo prime sequence
2R2D-1775KF1^a^	5′-GACT***GGTACC***ACCTAACTTGGTGCCTTTAG-3′
2R2D-1245KF5^a^	5′-GAAT***GGTACC***AGCTTGCTGTGTAGATG-3′
2R2D-540KF6^a^	5′-GACT***GGTACC***CATCATTATCAGTGGTGG-3′
2R2D+61HR3^a^	5′-GACT***AAGCTT***GTCTCCTGTTGCAAGAAGATC-3′
2R2D-1077RS1^b^	5′-AACAACTAAATGGCTATTGT-3′
2R2D-1245FS2^b^	5′-AGCTTGCTGTGTAGATGT-3′
2R2D-751FS3^b^	5′-GTCTGTAATTTAATAGTTGGTC-3′
NF1/B+536XF7^a^	5′-GAAT***CTCGAG***ATGATGTATTCTCCCATCTGTCTCAC-3′
NF1/B+2017ER8^a^	5′-GAAT***GAATTC***GCCCAGGTACCAGGACTG-3′
pEGFP-FS2p^b^	5′-AATGTCGTAACAACTCCGCCC-3′
pEGFP-RS2p^b^	5′-CGCTGAACTTGTGGCCGTTTA-3′

The primers for PCR were designed according to the published genomic sequences (NCBI gene ID No. 55844 for *PPP2R2D*, and NCBI gene ID No. 4781 for *NFI/B*).

a Primers used for target fragments' PCR and for plasmid construction. The bold italic letters refer to the restriction enzyme recognition sites.

b Primers used for sequencing.

### Linkage disequilibrium analysis and haplotype construction

The Hardy-Weinberg equilibrium (HWE) and pairwise linkage disequilibrium (LD) indices were calculated with the Haploview version 3.2 software (Broad Institute). We estimated the degree of pairwise LD between variants as quantified by the disequilibrium coefficients D′ or r^2^ [19]. They define pairs to be in ‘strong LD’ if the one-sided upper 95% confidence bound of D′ is >0.98 (that is, consistent with no historical recombination) and the lower bound is above 0.7. A *P* value of <0.05 was used as the criterion of statistical significance. All relevant haplotypes in the current population were also generated with Haploview.

### Plasmids construction

To evaluate the promoter activity of different parts of the 5′-flanking region of the *PPP2R2D* gene with various haplotypes, we made serial truncations of the *PPP2R2D* promoter fragment and analyzed the activity of the reporter constructs. Three different upstream primers and one downstream (+61HR3) primer (Table 1), flanked by restriction sites at the 5′-end, were designed for PCR reactions that employed human genomic DNA (wild-type) as template. The truncated fragments, which included F1 (−1775 nt to +61 nt, 1836 bp as distal promoter), F2 (−1245 nt to +61 nt, 1306 bp as middle promoter), and F3 (−540 nt to +61 nt, 601 bp as proximal promoter), were cloned into the pGL3-Basic Luciferase Reporter Vector (Promega), respectively. The recombinant plasmids, named pGL3b-2R2Dp-F1, -F2, and -F3, were confirmed by sequencing.

To examine the function of different haplotypes on *PPP2R2D* promoter activity, a series of reporter plasmids with different haplotypes encompassing the F1 fragments of human *PPP2R2D* were constructed by PCR from three genomic DNA samples with native haplotypes. The resulting constructs were termed pGL3b-2R2Dp-F1H1 (for distal 5′-flanking region F1, haplotype 1, and the rest follow in this manner), -F1H2, and -F1H3, each of which harbors 5′-flanking region with different haplotypes.

To study the effect of the −462 G>A variant on the transcriptional activity of the proximal promoter, reporter constructs containing either G or A variant were generated by subcloning from the PCR products of the genomic DNA samples that are heterozygous for −462 G>A variant (−462 GA heterozygote). The resulting two luciferase constructs with different allelotypes were designated as pGL3b-2R2Dp-462G and -462A, respectively. All fragments were digested with *Kpn* I and *Hind* III restriction enzymes and cloned in 5′-3′-orientation into a pGL3-Basic luciferase vector.

To construct the NF1/B expression plasmid (pEGFP-NF1/B), NF1/B coding sequence was amplified from total human cDNA by PCR using primers flanked with restriction sites (Table 1). The fragment was then cloned into the *Eco*R I and *Xho* I sites of pEGFP-N1 vector that contains an expression cassette of enhanced green fluorescent protein (EGFP). The pEGFP-N1 vector was purchased from Clontech (TaKaRa Bio Company). All reporter constructs and expression plasmids were further verified by direct nucleotides sequencing.

### Transient transfection and luciferase reporter assays

The immortalized human normal hepatocyte L02 cell line was kindly provided by Dr. Shi-mei Zhuang (Sun Yat-sen University) [20]. The L02 cells were maintained in RPMI-1640 medium (Thermo Fisher) supplemented with 10% fetal bovine serum (FBS, Thermo Fisher), 2 mM L-glutamine, 100 U/ml penicillin, and 100 µg/ml streptomycin (GIBCO) in a humidified incubator (37°C, 5% CO_2_).

For luciferase reporter assays, L02 cells (1×10^4^) were plated in 96-multiwell plates and grown to 80–90% confluence. Transfections were performed using Lipofectamine™ 2000 Reagent (Invitrogen) according to the manufacturer's instructions and a previous study from our group [18]. Cells were cotransfected with 50 ng of pRL-TK vector DNA (Promega) and 200 ng of either empty pGL3-Basic plasmid (a promoterless control; Promega) or one of several pGL3b-2R2Dp constructs harboring different lengths and allelotypes or haplotypes of the *PPP2R2D* promoter. The pRL-TK vector, which provided the constitutive expression of *Renilla* Luciferase, was cotransfected as an internal control to correct for differences in both transfection and harvesting efficiency. For cotransfection assays, various doses (250 ng, 500 ng, 1000 ng, respectively) of transcription factor NF1/B expression plasmid (pEGFP-NF1/B) or corresponding empty vector (pEGFP-N1), and reporter gene plasmids (200 ng of pGL3b-2R2Dp-462G or -462A, and 50 ng pRL-TK vector) were transfected into L02 cells. After 24 hours of incubation, cells were collected and analyzed for luciferase activity with the Dual-Luciferase Reporter Assay System (Promega). Promoter activity was reported as relative light units (RLUs) and normalized against the activity of the empty pGL3-Basic vector. All transfections were carried out in triplicate and repeated at least thrice in independent experiments.

### *In Silico* analysis of the allelic variants in 5′-flanking region *of PPP2R2D*

The genetic variants identified in this study were compared with data from the National Center for Biotechnology Information (NCBI) database (http://www.ncbi.nlm.nih.gov/dbSNP) and phase 3 data from the HapMap Public Release database (http://hapmap.ncbi.nlm.nih.gov/). All identified variants were then subjected to computer-aided analyses with the online program AliBaba 2.1 (http://www.alibaba2.com) to predict physical transcription factor binding sites (TFBS) in the given DNA sequence based on the TRANSFAC database of eukaryotic transcription factors (TFs; TRANSFAC10). The Transcription Element Search System (TESS) was also used to integrate potential TFBSs in the 5′-flanking region of *PPP2R2D* with various polymorphic alleles.

### Electrophoretic mobility shift assay (EMSA)

To investigate whether sequence variants alter DNA-protein interactions, EMSAs were performed with the *PPP2R2D* promoter fragment using the LightShift Chemiluminescent EMSA kit (Thermo Fisher), in accordance with the manufacturer's instructions. Nuclear proteins were extracted from confluent cultures of human L02 cells with a Nuclear Extraction Kit (Active Motif). Double-stranded oligonucleotides to be used as DNA probes were labeled with biotin at the 5′-end. Probe sequences corresponding to the −462 G>A variant in the *PPP2R2D* promoter region were 5′-biotin-GAATATTACATTGTACAGAAGCCAACAAAAGTTATTGCATAG-3′ for −462 G, and 5′-biotin-GAATATTACATTGTACAGAAACCAACAAAAGTTATTGCATAG-3′ for −462 A. The consensus sequence of the 5′-biotin-labeled double-stranded probe designed to recognize transcription factor NF1 was 5′-GGGCTAGATGGCTGCCAGCCAAGGCTTCAT-3′ [21]. All labeled and unlabeled oligonucleotides were ordered from Sangon Biotech Co., Ltd (Shanghai, China). Complementary oligonucleotides were annealed and used either as probe or competitor DNA. In each EMSA reaction, an aliquot of 5′-biotinylated double-stranded oligonucleotide (10 fmole) was incubated with 10 µg nuclear protein extract for 20 minutes at room temperature in 20 µl of binding buffer (100 mM Tris, 500 mM KCl, 10 mM DTT, pH 7.5) with 1 µl poly(dI•dC) (1 µg/µl). For competition experiments, a 200-fold molar excess of unlabeled cold probe was added to the mixture as a specific competitor before the incubation period. For the supershift experiment, 10 µg of nuclear extract was preincubated with anti-NF1 antibody (Santa Cruz Biotechnology Inc, CA) for 30 minutes at room temperature before the addition of labeled probe. After the addition of the labeled probe, the reactions were incubated for another 45 minutes on ice, then submitted to 6% native polyacrylamide gel electrophoresis, and transferred (0.5× TBE, 100 V, 60 minutes) onto a positively charged nylon transfer membrane (Magmaprobe, Osmonics Inc.). Bands were visualized by stabilized streptavidin-horseradish peroxidase conjugate.

### RNA extraction and real-time PCR

To analyze *PPP2R2D* mRNA levels, total RNA was extracted from L02 cell lines transfected with NF1/B encoding plasmids (250 ng, 500 ng, 1000 ng of pEGFP-NF1/B, respectively) or its corresponding empty vector (pEGFP-N1) using TRIzol® reagent (Invitrogen). Total RNA (500 ng) was reverse transcribed into signal-strand complementary DNA using oligo dT primer and PrimeScript™ RT Enzyme Mix Ι (SYBR® PrimeScript™ RT-PCR kit, TaKaRa). Relative gene expression quantitation for *PPP2R2D*, with *ACTB* as an internal reference gene, was performed with ABI 7500 system (Applied Biosystems) based on the SYBR-Green method. Primers used were: *PPP2R2D*, Forward, 5′-CGTGAACAAGAGAATAAAAGCCG-3′, Reverse, 5′-CTTCAATATTGGGACCCGTAG-3′; and *ACTB*, Forward, 5′-CACCAGGGCGTGATGGT-3′, Reverse, 5′-CTCAAACATGATCTGGGTCAT-3′. The PCR reaction mixture consisted of 0.1 µmol/L of each primer, 1× SYBR® *Premix EX Taq*™ (Perfect Real Time) premix reagent (TaKaRa), and 50 ng complementary DNA to a final volume of 20 µl. Cycling conditions were 95°C for 30 seconds, followed by 40 cycles at 95°C for 5 seconds and 60°C for 34 seconds. The averaged cycle threshold (C_T_) values of each reaction derived from the target gene, determined with ABI 7500 system software, were normalized to *ACTB* levels. The ΔC_T_ was calculated by subtracting the C_T_ of *ACTB* from the C_T_ of the target gene of interest. The ΔΔC_T_ was calculated by subtracting the ΔC_T_ of the reference sample from the ΔC_T_ of each sample. Fold change was generated using the equation 2^−ΔΔCT^.

### Western blot analysis

Total proteins were extracted from L02 cells that cotransfected with pEGFP-NF1/B plasmid with lysis buffer (50 mM HEPES pH 7.6, 250 mM NaCl, 0.1% NP-40, 5 mM EDTA, 0.5 mM PMSF and the proteinase inhibitor cocktail). 70 µg of total proteins were used for each lane of loaded sample. The protein blots were blocked with 5% fat-free milk in TBS buffer for 1 hour and then incubated for overnight at 4°C with antibody against PP2A-Bδ (Santa Cruz Biotechnology Inc, CA), and β-actin antibody (Cell Signaling Technology, MA) at a dilution of 1∶500 and 1∶3000, respectively. The secondary antibody was anti-goat IgG or anti-mouse IgG (Santa Cruz Biotechnology Inc, CA) at a dilution of 1∶1000 and 1∶3000 respectively. The blots were detected using the enhanced chemiluminescence method (Pierce).

### Statistical analysis

LD analysis was performed using Haploview. The genotype frequencies in the study subjects were compared with the frequencies expected by Hardy-Weinberg equilibrium (HWE) by goodness-of-fit *χ* ^2^, and the *χ* ^2^ values for statistical significance and their corresponding *P* values were calculated. Pairwise LD between SNPs was measured using the disequilibrium parameters D′ and r^2^. D is a measure of the difference between the frequency of a haplotype and the frequency that is expected under Hardy-Weinberg Equilibrium. D′ is a standardized version of D that ranges from 0 to 1. The higher the D′-value, the greater the association between the two loci being studied. Heterozygosity (π) was calculated as the average of 2jk/n(n-1) for all base pairs screened, with j and k referring to the number of copies of the minor and major alleles, respectively (n = j+k). Student's *t* test for two groups or ANOVA with an *S-N-K* test for multiple groups was used to examine the differences in luciferase reporter gene expression. *P*<0.05 was used as the criterion for statistical significance. All statistical tests were two-sided.

## Results

### Identification of genetic variants within the 5′-flanking region of *PPP2R2D* in the southern Han Chinese population

**Figure 1:**
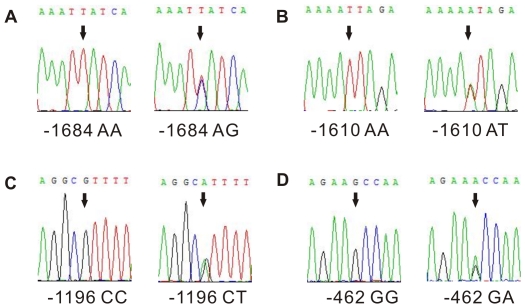
The four identified variants in the 5′-flanking region of *PPP2R2D*. The 5′-flanking fragment containing 1836 bp (−1775 nt to +61 nt) around the transcription start site (TSS) was amplified by PCR and resequenced with an ABI 3730 automatic sequencer. The genomic DNA samples collected from 70 healthy donors in southern Han Chinese population. The direct DNA resequencing revealed 4 genetic variations located in this 5′-flanking region of *PPP2R2D*. **A.** were the sequences for −1684 AA and −1684 AG genotypes; **B.** were the sequences for −1610 AA and −1610 AT genotypes; **C.** were the sequences for −1196 CC and −1196 CT genotypes; **D.** were the sequences for −462 GG and −462 GA genotypes. The arrows indicated the nucleotide variants.

We sequenced a 1836 bp segment of the 5′-flanking region of *PPP2R2D* in seventy healthy subjects and identified four genetic variants (Table 2). The four variants found in this population are registered in the current NCBI SNP database (dbSNP): −1684 A>G (rs11146169) (Fig. 1A), −1610 A>T (rs77523588) (Fig. 1B), −1196 C>T (rs9419202) (Fig. 1C), and −462 G>A (rs7074421) (Fig. 1D). Although −1684 A>G (rs11146169) is present in the dbSNP database, the minor allele frequency (MAF) for this site is less than 1% in our study population. Among the seventy healthy Han Chinese subjects, the MAF for the four genetic variants in all the variant allelotypes was −1684 G (0.71%), −1610 T (5.71%), −1196 T (2.14%), −462 A (2.14%). All the allele frequencies were in HWE (*P*>0.05). The MAFs for three of the variants (−1684 A>G, −1196 C>T, and −462 G>A) were less than 5%. Not surprisingly, none of the minor alleles of the three variants were observed in the homozygous state in any of the samples in the current population. Information regarding the SNP coordinates, MAF, and HWE of the variants is presented in Table 2.

**Table 2:** The characteristics of the 4 variations identified in the 5′-flanking region of *PPP2R2D* in 70 southern Han Chinese subjects.

Genetic variants	dbSNP ID no.	Location^a^	Chr. position^b^	MAF^c^	HWE test *(P)* d	TRANSFAC prediction^e^
−1684 A>G	rs11146169	5′ near gene	133746276	0.71(G)	0.952	+AP1,+HNF1, −Oct-11
−1610 A>T	rs77523588	5′ near gene	133746350	5.71(T)	0.612	−C/EBPα, −HOXA4, +MEF-2
−1196 C>T	rs9419202	5′ near gene	133746764	2.14(T)	0.855	−Sp1, −Oct-1
−462 G>A	rs7074421	5′ near gene	133747498	2.14(A)	0.855	−NF1, −HNF-3

a Location relative to the transcription start site (TSS).

b Chr. position = nucleotide numbering according to GeneBank ID No. 55844.

c MAF: minor allele frequency (%) in the studied population.

d HWE = Hardy-Weinberg equilibrium with *χ* ^2^ test.

e + = predicted gain of a transcription factor binding site. −  = predicted loss of a transcription factor binding site.

To characterize the distribution of these variants in the 5′-flanking region of *PPP2R2D*, the MAFs in this southern Han Chinese population were compared to those in HapMap populations. The four genetic variants have been observed previously in various populations and their allele frequencies (from HapMap phase 3 data) are reported in Table 3. The MAF of −1684 A>G in our sample (0.71%, n = 140) was lower than that reported for the Asian populations in HapMap-HCB (2.22%, n = 90) and HapMap-JPT (1.11%, n = 90), but the frequencies were not statistically different from each other (*P*>0.05). The MAF of −1610 A>T in our study (5.71%) was similar to that of Asian populations in HapMap-HCB and -JPT (5.0%, n = 120). The MAF of −1196 C>T in our study (2.14%, n = 140) was identical to that in HapMap-HCB (1.2%, n = 86) and HapMap-JPT (2.3%, n = 172) (*P*>0.05). However, in the HapMap-CEU (14.6%, n = 226) and HapMap-YRI (21.7%, n = 226) populations, the MAFs of −1196 C>T were significantly higher than the MAF of this variant in the Han population of this study (*P*<0.05). The MAF of −462 G>A in our study (2.14%, n = 140) was distributed similarly to the −1196 C>T. Taken together, these results indicate that there are significant differences in the genotypic distributions of the −1196 C>T and −462 G>A variants between our southern Han Chinese group and European/sub-Saharan African groups in HapMap.

**Table 3:** Comparison for MAF of variants in the 5′-flanking region of *PPP2R2D* between southern Han Chinese and HapMap populations.

Variants	Current studied population	HapMap-HCB^a^	HapMap-JPT^a^	HapMap-CEU^a^	HapMap-YRI^a^
−1684 A>G	0.71% (1/140)	2.2%(2/90) (*χ* ^2^ = 0.151, *P* = 0.698)	1.1%(1/90) (*χ* ^2^ = 0.000, *P* = 1.000)	—b	—b
−1610 A>T	5.71% (8/140)	5.0%(6/120)^d^ (*χ* ^2^ = 0.065,*P* = 0.79) (HapMap-HCB+JPT)		—c	—c
−1196 C>T	2.14% (3/140)	1.2%(1/86) (*χ* ^2^ = 0.001, *P* = 0.982)	2.3%(4/172) (*χ* ^2^ = 0.000, *P* = 1.000)	14.6%^d^(33/226) (*χ* ^2^ = 15.131, *P* = 0.000)	21.7%^d^(49/226) (*χ* ^2^ = 27.075, *P* = 0.000)
−462G>A	2.14% (3/140)	1.2%(1/86) (*χ* ^2^ = 0.001, *P* = 0.982)	2.3%(4/172) (*χ* ^2^ = 0.000, *P* = 1.000)	14.6%^d^(33/226) (*χ* ^2^ = 15.131, *P* = 0.000)	21.7%^d^(49/226) (*χ* ^2^ = 27.075, *P* = 0.000)

a Data from HapMap database (http://www.hapmap.org) showing the MAF in various populations, including HCB, JPT, CEU, YRI. Abbreviations: HCB: Han Chinese in Beijing, China; JPT: Japanese in Tokyo, Japan; CEU: Utah residents with Northern and Western European ancestry from the CEPH collection; YRI: Yoruban in Ibadan, Nigeria.

b . The data display no G allele.

c . No published data in HapMap database.

d . Statistic difference between current studied population and HapMap populations with *χ* ^2^ test (*P*<0.05).

Next we assessed the degree of heterozygosity (π) in the southern Han population with HapMap phase 3 data. The heterozygosity of −1684 A>G (π = 0.014, n = 140) in the Han Chinese population was found to be slightly lower than that in the HapMap-HCB (π = 0.044, n = 90) and HapMap-JPT (π = 0.022, n = 90) populations, but showed no significant difference. The π value for −1610 A>T (π = 0.109, n = 140) was similar to that in HapMap-HCB+JPT (π = 0.096, n = 120) (*P*>0.05). The π value for −1196 C>T (π = 0.042, n = 140) was also found to be similar to that in HapMap-JPT (π = 0.046, n = 172) and slight higher than that in HapMap-HCB (π = 0.023, n = 86) but displayed no significant difference. However, the π values of −1196 C>T (π = 0.042, n = 140) was significantly lower than that in HapMap-CEU (π = 0.251, n = 226) and HapMap-YRI (π = 0.341, n = 226) populations (*P*<0.01). The π value for −462 G>A (π = 0.042, n = 140) was distributed similarly to the −1196 C>T. These results indicate that the differences in heterozygosity might be due to racial differences. Variations with high heterozygosity values usually have functional significance and merit further study.

### Linkage disequilibrium (LD) and haplotype analysis

**Figure 2:**
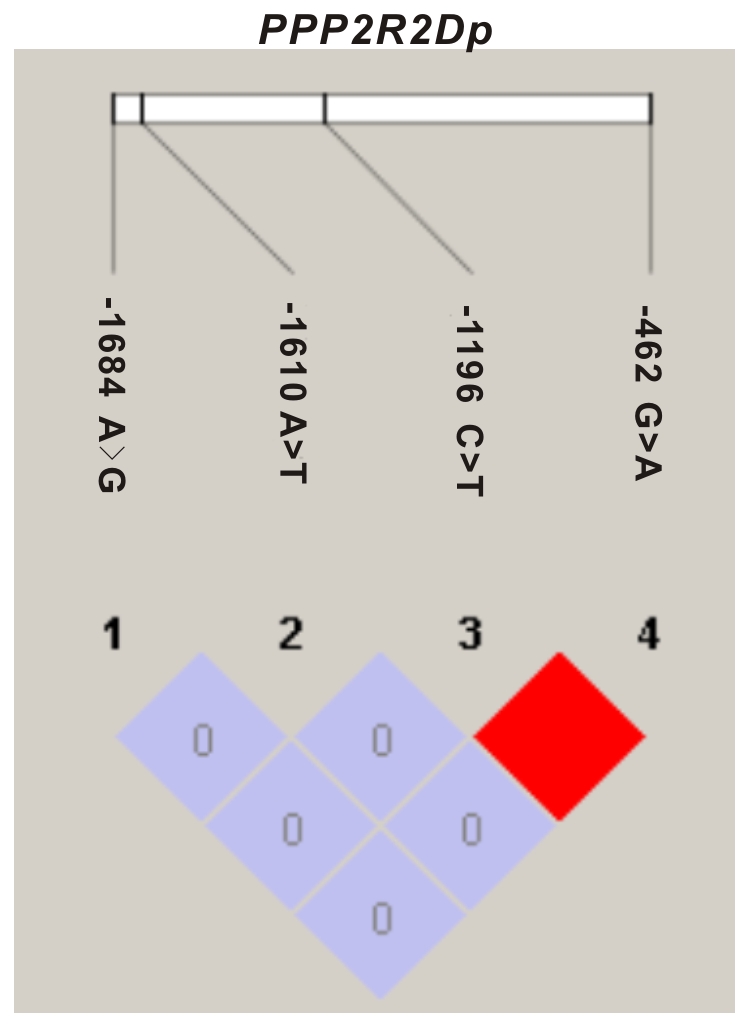
The LD structure of genetic variations in the 5′-flanking region of *PPP2R2D*. Pairwise linkage disequilibrium (LD) values between variants were calculated using Haploview in current Han Chinese population. The relative locations of variants are indicated along the top. The value within each diamond represents the pairwise correlation between variant sites (measured as r^2^) defined by the upper left and the upper right sides of the diamond. The diamond without a number corresponds to D′ = 1. Red diamonds indicate statistically significant allelic association between the pairs of variations, as measured by D′; Blue diamonds indicate pairwise D′ values of 1, but without statistical significance.

To examine the degree of linkage among the four variants identified in the 5′-flanking region of *PPP2R2D*, We quantified the degree of pairwise linkage disequilibrium (LD) using the disequilibrium coefficient D′ or r^2^ and the EM-based algorithm of Haploview 3.2. Because there is no single best measure of LD that is universally applicable, the complementary measures D′ and r^2^ were used in this study [22]. The −1196 C>T and −462 G>A variants were found to be in high LD in this Han Chinese population, with r^2^ = 1.0 and the lower and upper bounds of the 95% confidence interval (95% CI) for D′ at 0.59 and 1.00, respectively (Fig. 2). However, other variants were found to be in low LD, with an upper 95% CI bound for D′ of 0.97 and a lower bound of 0.04 (r^2^<0.01) in this population sample. The LD results are consistent with the distribution of genotypes and the heterozygosity of these genetic variants in the southern Han Chinese population.

Haplotype analysis was performed on variants in the 5′flanking region (−1775 nt to +61 nt; F1 fragment) of *PPP2R2D* to identify haplotypes among the southern Han Chinese population and to estimate their frequencies. Three major novel haplotypes (F1H1, F1H2, and F1H3) were identified and their frequencies were calculated in our sample population; the other haplotype identified was rare and accounted for 0.72% (Table 4). Consistent with the LD analysis described above, alleles −1196 C and −462 G were found to be transmitted together, as were the −1196 T and −462 A alleles. However, no separate haplotype blocks were observed might help to suggest that the different 5′-flanking regions are independent functional domains in *PPP2R2D* gene. Tag SNPs were found for three of the variants (−1684 A>G, −1610 A>T, and −462 G>A) in the distal and proximal 5′-flanking region of the *PPP2R2D* in current study sample.

**Table 4:** Haplotypes formed by genetic variants in the distal promoter of *PPP2R2D*.

Haplotypes^a^	−1684 A>G	−1610 A>T	−1196 C>T	−462 G>A	Frequency (%)
F1H1	A	A	C	G	92.14
F1H2	A	T	C	G	5.00
F1H3	A	A	T	A	2.14
Other					0.72
Total					100.0

a Haplotype analysis was performed in the distal promoter region (−1775 nt to +61 nt) of *PPP2R2D* with Haploview. The haplotypes (F1H1 - F1H3, F1H1 was for the distal promoter with haplotype 1, and the rest following in this manner) were identified with different alleles in 4 variants. The frequencies of each haplotype were calculated in current southern Han Chinese population.

### Promoter activity of the 5′-flanking region of *PPP2R2D*

**Figure 3:**
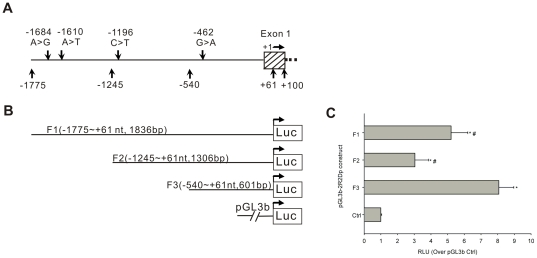
Scheme of the 5′-flanking region of *PPP2R2D* and identification of the active core promoter. **A.** Human *PPP2R2D* 5′-flanking region identified in the NCBI database. The box with shadow represents exon 1. The four variants identified in current study, −1684 A>G (rs11146169), −1610 A>T (rs77523588), −1196 C>T (rs9419202), and −462 G>A (rs7074421), are located in the 5′-flanking region (−1775 nt to +61 nt) of *PPP2R2D*. The *PPP2R2D* genomic reference sequence with GeneBank ID no. 55844 was used with the first nucleotide of the RNA transcript as +1 nt. Other numbers represent primer positions for cloning reporter constructs. **B.** Fragments F1, F2, and F3 were amplified by PCR to make reporter constructs, and their positions and lengths are shown in parentheses. Each fragment with wild-type alleles was cloned into the pGL3-Basic vector. **C.** Relative luciferase activity of series truncated constructs of 5′-flanking region of *PPP2R2D* in human L02 cells. Each construct or empty vector was transfected into L02 cells with pRL-TK plasmid as an internal intrinsic control of transfection efficiency. After 24 hours, the luciferase activities from each testing construct and also from the internal control plasmid were measured using Dual-Luciferase Reporter Assay System. RLU indicates relative light units. The relative luciferase activity was normalized to *Renilla* activity and was relative to pGL3-Basic control (Ctrl), which was set as 1.0 RLU. Compared with the basal activity of Ctrl, the promoter activity of all constructs was increased at statistically significant levels (*P<0.05). Compared with the activity of the F3 construct, all constructs showed significant lower activity (**^#^** *P*<0.05). The results represent the mean ± SD of three independent experiments.

We next attempted to determine whether this fragment (−1775 nt to +61 nt) constitutes an active promoter. We amplified a 1836 bp fragment containing the 5′-flanking region of *PPP2R2D* using wild-type human genomic DNA as the template (Fig. 3A). Subsequently, we generated truncated constructs (F1, F2, and F3) by progressive deletion of nucleotides from the 5′-end and cloned these fragments into the pGL3-Basic luciferase vector (Fig. 3B). These constructs were transiently transfected into human L02 cells and tested for luciferase activity to define the minimal sequence for the transcription of *PPP2R2D*.

As shown in Fig. 3C, the promoter activity of all the constructs was higher than the basal activity of the pGL3-Basic vector used as control (Ctrl) (*P*<0.05). Sequential deletions made to the 5′-end of the 1836 bp F1 fragment to generate the construct from F1 to the 1306 bp F2 fragment resulted in a decrease in luciferase activity of approximately 42% (relative to the longest construct, F1). This result indicates that transcriptional repression exists within this distal promoter region. Removal of 705 bp from the 5′-end of the F2 fragment generated construct F3 (−540 nt to +61 nt). F3 generated 125% of the luciferase activity of the F1 construct and 213% of the activity of the F2 construct. This indicates that critical *cis*-acting elements required for *PPP2R2D* transcription are located in the F3 region harboring the proximal promoter and suggests that the region between −540 nt and +61 nt is responsible for most of the promoter activity of *PPP2R2D*. Therefore, the F3 fragment of the proximal 5′-flanking region of *PPP2R2D* is recognized as the active core promoter. The F3 fragment contains one of the four variants identified earlier (−462 G>A). To investigate the effect of this potential functional SNP on the expression of PP2A-Bδ, the 5′-flanking region of *PPP2R2D* containing the −462 G or −462 A allele was subjected to further functional analysis for the promoter activity.

### Differential proximal promoter activity of *PPP2R2D* with −462 G>A variants

**Figure 4:**
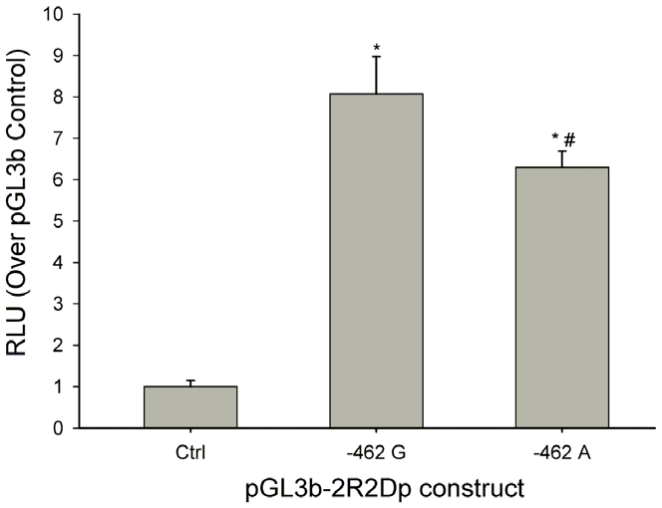
Functional analysis for −462 G>A in *PPP2R2D* proximal promoter. Transcriptional activity of F3 fragment bearing *PPP2R2D* proximal promoter with variant allelotype of −462 G or −462 A was measured by Dual Luciferase Assays. Results are expressed as fold increases in relative light units (RLU) over the empty pGL3-Basic vector (Ctrl). Compared with the basal activity of Ctrl, the promoter activity of the pGL3b-2R2Dp-462G or −462A was higher (*P<0.05). Construct with the −462 G allele had higher activity compared with the −462 A allele (**^#^** *P*<0.05). The results represent the mean ± SD of three independent experiments.

We wished to assess the impact of the −462 G>A variant on the transcriptional activity of the *PPP2R2D* proximal promoter. We generated two luciferase reporter gene constructs, pGL3b-2R2Dp −462 G and −462 A, that contained different alleles at position −462 nt in the proximal promoter region of *PPP2R2D*. The recombinant plasmids were transfected into L02 cells. The empty vector (pGL3b) and a *Renilla* construct (pRL-TK) were used as controls (Ctrl). As shown in Fig. 4, the two *PPP2R2D* proximal promoter constructs generated higher levels of luciferase expression than the empty vector control (*P*<0.05). The two constructs also differed from each other in luciferase expression. The F3 fragment containing variant −462 G displayed the higher transcriptional activity than the fragment containing −462 A (*P*<0.05). These data indicate that different allelotypes have a significant effect on the transcriptional activity of the *PPP2R2D* proximal promoter.

### Differential transcriptional activity of different *PPP2R2D* promoter haplotypes

**Figure 5:**
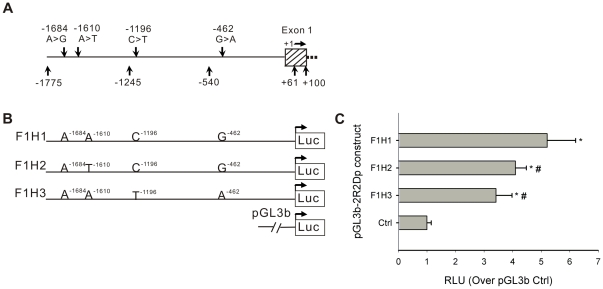
Transcriptional activity of the *PPP2R2D* promoter with variant haplotypes. **A.** Human *PPP2R2D* 5′-flanking region identified in the NCBI database. These four variants, −1684 A>G (rs11146169), −1610 A>T (rs77523588), −1196 C>T (rs9419202), and −462 G>A (rs7074421), are located in the distal promoter of *PPP2R2D* (−1775 nt to +61 nt). **B.** F1 fragment harboring different haplotypes (F1H1, F1H2, and F1H3) were amplified by PCR to make reporter constructs, and their various haplotypes are shown above the line. Each fragment was cloned into the pGL3-Basic vector and transfected into L02 cells. **C.** Transcriptional activities of *PPP2R2D* promoter with various haplotypes (F1Hs) were measured by Dual Luciferase Assays. Results are expressed as fold increases in relative light units (RLU) over the empty pGL3-Basic vector (Ctrl). Compared with the basal activity of Ctrl, the promoter activity of all the F1Hs was higher (*P<0.05). Compared with the F1H1 activity, F1H2 and F1H3 showed significant lower activity (**^#^** *P*<0.05). The results represent the mean ± SD of three independent experiments.

To assess the impact of the potentially functional variant identified above (Fig. 5A) on the transcriptional activity of the different *PPP2R2D* promoter haplotypes, we generated three luciferase reporter gene constructs (pGL3b-2R2Dp-F1H1 — -F1H3), each of which contains a different haplotype in the promoter region of *PPP2R2D* (Fig. 5B). The recombinant plasmids were transfected into L02 cells along with the empty vector (pGL3b) and a *Renilla* construct (pRL-TK), which were used as controls. As shown in Fig. 5C, all the constructs provided higher levels of luciferase expression than the basal activity (Ctrl) of the empty vector (pGL3b) (*P*<0.05). However, the haplotype constructs differed from each other in their transcriptional activity. The F1H1 and F1H2 fragments containing the −462 G variant showed 52.4% and 20.2% higher transcriptional activity than the F1H3 fragment containing −462 A (*P*<0.05). Given that the region between −540 nt and +61 nt is the core promoter, the −462 G>A variant may be a functional variant involved in the transcriptional regulation of the *PPP2R2D* gene. Although both the F1H1 and F1H2 haplotypes contain −462 G allelotype, the transcriptional activity of the H2 haplotype was 21.3% less than that of H1. This indicates that no single variant completely accounts for the expression differences observed at the haplotype level. Taken together, these data indicate that different variants associated with the functional −462 G>A variant in the haplotypes play significant roles in the transcriptional activity of the *PPP2R2D* promoter.

### The −462 G>A variant affects NF1 binding to the proximal promoter of *PPP2R2D*

*In Silico* analysis of the 5′-flanking region of *PPP2R2D* using a computer algorithm revealed several putative binding sites for transcriptional factors (TFs). Some of TFs binding patterns changed when the observed genetic variation in 5′-flanking region of *PPP2R2D* was taken into account (Table 2). Interestingly, the −462 G>A variant located in the proximal promoter of *PPP2R2D* altered the binding site of the transcription factor NF1 (Table 2). To further confirm this finding, we performed an electrophoretic mobility shift assay (EMSA).

**Figure 6:**
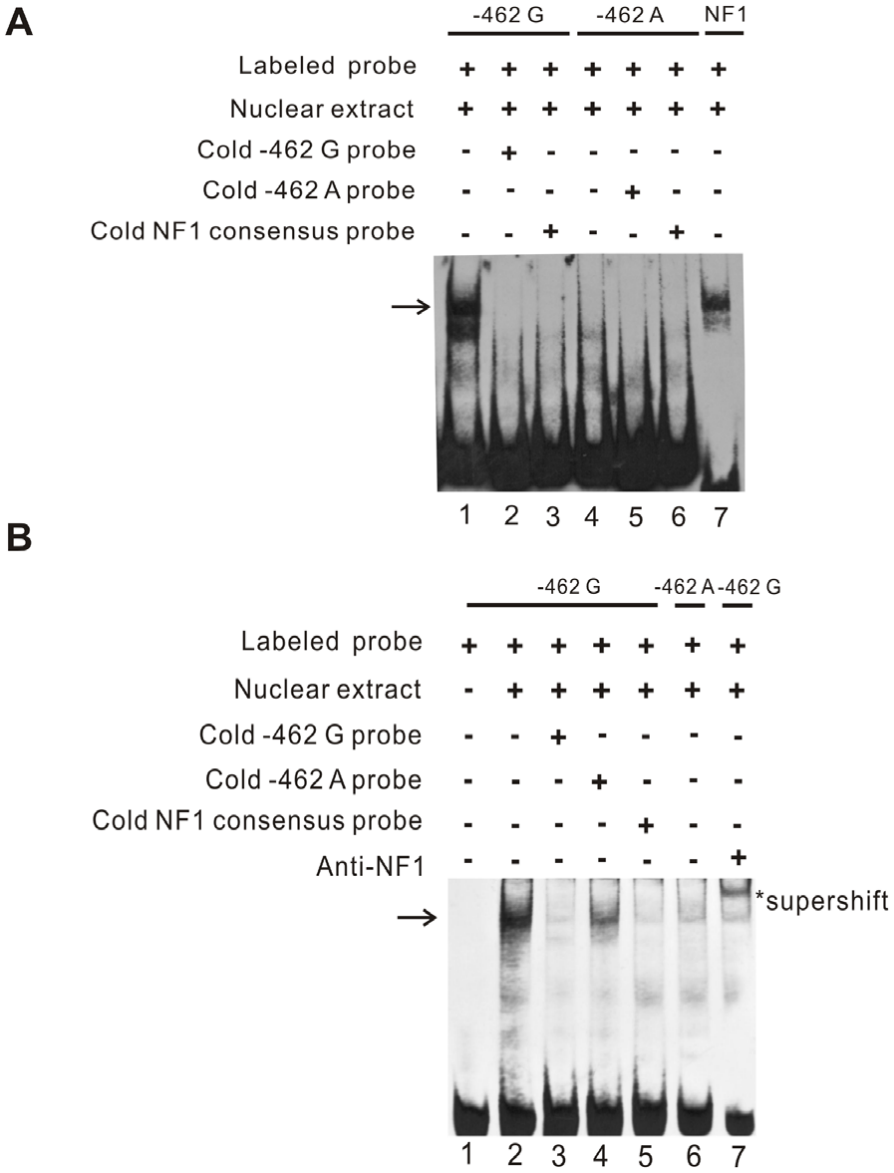
EMSA of *PPP2R2D* core proximal promoter region containing the −462 G>A variation. **A.** 5′-end biotin-labeled double-stranded oligonucleotides probes including −462 G (lane 1–3) or −462 A (lane 4–6) were incubated with 10 µg of nuclear protein extracted from human L02 cells. The DNA-protein complex (indicated by arrow) showed difference between the −462 G and −462 A allele. For competition EMSA, a 200-fold molar excess of unlabeled/cold −462 G probe (lane 2) or −462 A probe (lane 5) was included in the binding reactions as specific competitors. A 200-fold molar excess of unlabeled consensus NF1 oligonucleotides (lane 3 and 6) were used as competitors to determine the critical binding site. Labeled consensus NF1 probe was included as a positive control (lane 7). **B.** For cross-competition and supershift assays, a 200-fold molar excess of unlabeled/cold −462 G probe (lane 3), −462 A probe (lane 4) or consensus NF1 oligonucleotides (lane 5) or anti-NF1 antibody (lane 7) was preincubated 30 minutes with nuclear protein. The arrow indicates the DNA-protein complex. The asterisk indicates the supershifted complexes. The sequences of all probes used in the experiment were shown in methods.

To identify whether these *cis*-acting elements physically interact with *trans*-factors, we incubated nuclear extracts from human L02 cells with two 5′-biotinylated double-stranded oligonucleotides containing the −462 G or the −462 A variant. In mobility shift assays where only one band was observed on the gel, a DNA-protein complex was detected with the −462 G probe, but not with the −462 A probe (indicated by the arrow in Fig. 6, A and B). The difference in the binding affinity of these two probes was shown by competition experiments using unlabeled −462 G or −462 A cold probes. The DNA-protein complex containing the −462 G probe (Fig. 6A, lane 1) was completely outcompeted by the addition of a 200-fold molar excess of unlabeled −462 G probe (Fig. 6A, lane 2). Besides, the DNA-protein complexes in lanes 1 (Fig. 6A) are in the same position as the labeled NF1 consensus probe (Fig. 6A, lane 7), suggesting that NF1 in the nuclear extract bound to the probe.

Consistent with the competition assay, the cross-competition assay showed that this DNA-protein complex was completely outcompeted by the same amount of unlabeled NF1 consensus probe (Fig. 6A, lane 3; Fig. 6B, lane 5), but not outcompeted by the same amount of unlabeled −462 A probe (Fig. 6B, lane 4), suggesting that the protein bound to the −462 G probe may be NF1 and the −462 G probe has specific affinity for nuclear proteins. Supershift assay was performed at the same time to determine whether NF1 could bind this sequence containing −462 G variant. As shown in Fig. 6B, the presence of anti-NF1 antibody in the sample containing nuclear protein and labeled −462 G probe resulted in a clear supershift complex of the band (Fig. 6B, lane 7). These results indicate that the presence of −462 G or −462 A in the proximal promoter region of *PPP2R2D* may affect the binding of NF1 to its target region in a sequence-specific manner.

### NF1 plays an important regulatory role in PP2A-Bδ gene expression

**Figure 7:**
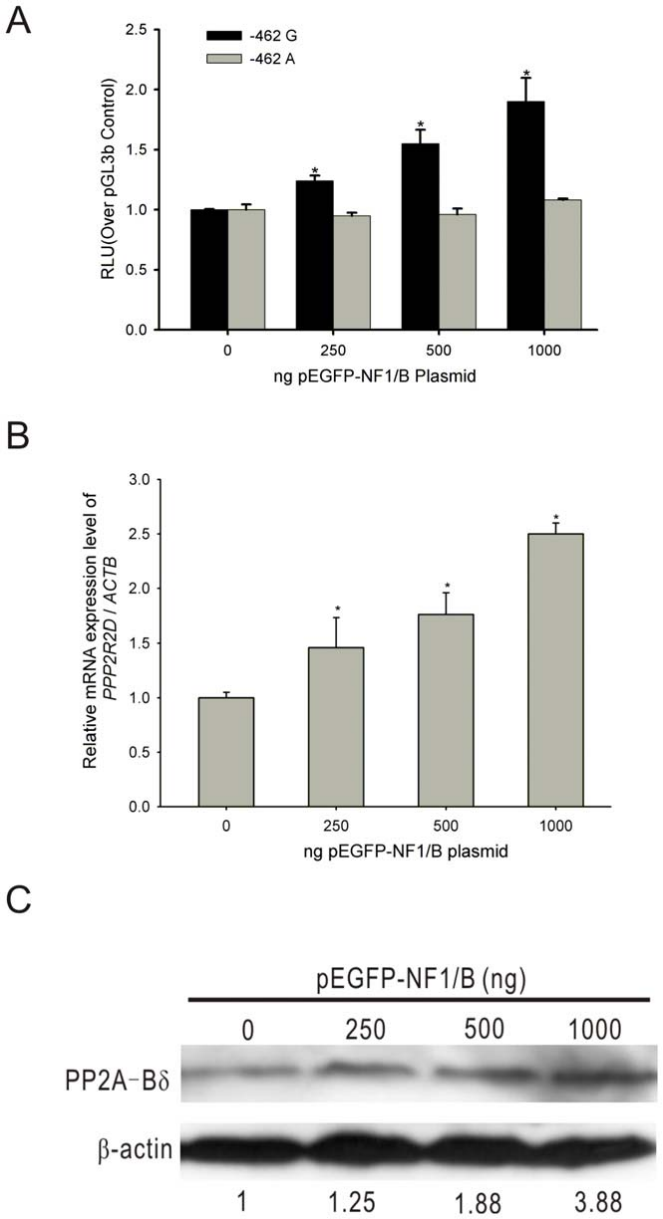
Effects of NF1 on the transcriptional activity and gene expression of *PPP2R2D*. **A.** Expression of the exogenous NF1/B in L02 cells induced dose-dependent increase in the luciferase reporter activity within 250 ng to 1000 ng of the pEGFP-NF1/B plasmid (*P<0.05). **B.** Transfection of pEGFP-NF1/B or its corresponding empty vector (pEGFP-N1) into L02 cells and cells were harvested 24 hours after transfection. *PPP2R2D* transcripts were measured and normalized to *ACTB* by real-time RT-PCR. The relative ratio of *PPP2R2D* mRNA in L02 cells transfected with empty vector was set as 1.0 for a control. The relative expression levels of the endogenous *PPP2R2D* were increased in a dose-dependent manner after introduction of 250 ng to 1000 ng pEGFP-NF1/B plasmid (*P<0.05). **C.** Overexpression of the exogenous NF1/B in L02 cells induced dose-dependent enhanced expression of the endogenous PP2A-Bδ (*P<0.05). The results represent the mean ± SD of three independent experiments.

Next, we designed cotransfection experiments in L02 cell to provide direct evidence of identified transcription factor NF1 regulating *PPP2R2D* gene promoter activity and PP2A-Bδ expression. As shown in Fig. 7A, overexpression of the NF1/B induced a dose-dependent increase in the luciferase activity of −462 G luciferase reporter in L02 cells. However, this dose-dependent response was abolished in −462 A luciferase reporter. Furthermore, we also detected the *PPP2R2D* mRNA and protein expression levels of L02 cells (−462 GG genotype) transfected with NF1/B encoding plasmids by using real-time RT-PCR and western blot analysis. As shown in Fig. 7B and Fig. 7C, the mRNA and protein expression levels of endogenous *PPP2R2D* were enhanced in a dose-dependent manner after overexpression of 250 ng to 1000 ng exogenous NF1/B expression plasmid. Together, these results demonstrate that NF1/B displays a positive regulation on the transcription of *PPP2R2D* gene and upregulates the expression of PP2A-Bδ.

## Discussion

The results of this study demonstrate, for the first time, that genetic variants and their haplotypes in the 5′-flanking region of *PPP2R2D* gene can contribute to variations in the transcriptional regulation of PP2A-Bδ. The identified four known genetic variants, three haplotypes in the promoter region and their distribution in the population provide fundamental information for genotyping *PPP2R2D* in the southern Han Chinese population. Experimental data indicate that the functional variant −462 G>A involves in the transcriptional regulation of *PPP2R2D* and the transcription factor NF1 interacts with the −462 G>A variant present within the proximal promoter region of *PPP2R2D*. Moreover, promoter fragments harboring different haplotypes exhibit different promoter activities, which imply that genetic variants are likely to regulate PP2A-Bδ expression and, consequently influence its dephosphorylation functions.

Protein phosphatase 2A (PP2A) is one of the most abundantly expressed serine/threonine protein phosphatases. A large body of evidence suggests that PP2A is a tumor suppressor [23], [24], [25], and that it plays critical roles in the regulation of many cellular processes [26], [27], [28]. The regulation of regulatory B subunits of PP2A (PP2A-Bs) ensures that dephosphorylation of specific substrates occurs in distinct cellular compartments and within specific signal transduction pathways [8], [29], [30]. The functions of B family subunit (PR55, B55) include the regulation of cytoskeletal dynamics and mitogen-activated protein kinase (MAPK) signaling, and apoptosis [10], [31], [32], [33]. Silencing of the B-family regulatory subunit, Bδ, leads to hyperactivation of the extracellular signal-regulated kinase (ERK) caused by constitutively active MEK1. To date, there have been no publications characterizing the regulatory regions of the human *PPP2R2D* gene. More importantly, little work has been done to investigate potentially functional genetic variants in the 5′-flanking region of the regulatory Bδ subunit gene and characterize their distribution in different populations. In order to gather more information about regulatory polymorphisms (rSNPs) in the 5′-flanking region of the gene coding for PP2A-Bδ, we conducted a functional analysis of the 5′-flanking region of *PPP2R2D*.

Firstly by analyzing the variant characteristics in target population, we found that these four genetic variants, in the 5′-flanking region (−1775 nt to +61 nt) of the *PPP2R2D*, differ greatly in frequency from those in other ethnic populations in the HapMap phase 3 database. In this southern Han population, the allele frequencies of the −1684 A>G, −1610 A>T, −1196 C>T, and −462 G>A variants were determined to be 0.993∶0.007, 0.943∶0.057, 0.979∶0.021, and 0.979∶0.021, respectively; the minor G, T, T, and A alleles (for −1684 A>G, −1610 A>T, −1196 C>T, and −462 G>A, respectively) are rare and only heterozygous genotypes were found in this population. Interestingly, our study shows that the MAFs of the variants differ between our study population and the Han Chinese in Beijing, China. This is consistent with a previous study showing that the greatest genetic differences among the Han Chinese are between the northern Han Chinese (N-Han) and southern Han Chinese (S-Han) [34]. Moreover, studies based on analyses of archeological, anatomical, linguistic, and genetic data have consistently suggested the existence of a significant boundary between the northern and southern populations in China [35]. These results suggest that genetic variants differ in their occurrence across ethnic groups. Our data not only update the HapMap database, but they also reveal significant differences between different ethnic subgroups.

SNPs and their characteristic distribution in a target population are valuable resources for investigating the genetic basis of individual susceptibility to damage or even disease. These variants can now serve as markers for fine-scale genetic mapping experiments and genome-wide association studies (GWAS) [36]. Both tumorigenicity and functional haploinsufficiency have been attributed to mutations in the A subunit of PP2A (PP2A-A). These mutations promote degradation of A subunit [37], [38]. With regard to the B subunits of PP2A (PP2A-Bs), a recent study demonstrated that a SNP in *PPP2R5E* (the gene coding for B56ε, a B56 family regulatory subunit of PP2A) is associated with human soft tissue sarcoma [39]. Furthermore, a case-control study using haplotype analysis of tagged SNPs in the *PPP2R2A* (coding for PP2A-Bα) and *PPP2R1A* (coding for PP2A-Aα) genes demonstrated that certain genotypes were protective for breast cancer, while others modified the risk for women with proliferative breast lesions [40]. A candidate gene association study identified a SNP (rs319217, A>G) in the *PPP2R2B* gene (coding for PP2A-Bβ) that manifests allelic differences in the cellular responses to treatment with chemotherapeutic agents [41]. In addition, a study suggested that a CAG repeat polymorphism in the *PPP2R2B* gene may be functional and may, in part, play a role in conferring susceptibility to Alzheimer's disease (AD) and essential tremor (ET) in Taiwan [42]. A similar study from Kimura *et al.* suggested that CAG repeat lengths in the *PPP2R2B* gene may be potential genetic markers for AD susceptibility in the Japanese population [43]. Lin and colleagues have reported that the CAG repeat length, in addition to the CREB, SP1, and TFAP4 proteins, plays a critical role in regulating *PPP2R2B* promoter activity [44]. More recently, a study indicated that the A variant of a SNP (rs319217 A>G) in the *PPP2R2B* gene is a marker for improved prognosis of breast cancer [45]. However, the genetic variants in the *PPP2R2D* gene remain largely unknown. In the light of previous reports implicating PP2A-Bδ in the regulation of the cell cycle and the dephosphorylation of specific substrates, we designed experiments aimed at functionally analyzing the identified variants in the 5′-flanking region of *PPP2R2D*.

We designated a region of 601 bp (−540 nt to +61 nt) as the core proximal promoter of *PPP2R2D* because it displayed the highest transcriptional activity in luciferase assays. We constructed proximal promoter fragments mirroring the *PPP2R2D* allelotypes, including either one or the other allele of the −462 G>A variant, and tested them for transcriptional activity. Promoter activity assay revealed that the most common allelotype (F3H1), containing the G^−462^ polymorphism, exhibited a higher transcriptional activity. The other construct bearing A^−462^ displayed a lower level of promoter activity. Our results suggest that different allelotypes composed of the −462 G>A variant influence the transcriptional activity of *PPP2R2D*.

To determine whether the transcription of *PPP2R2D* was regulated by different haplotypes, we constructed the distal fragments (F1) in the 5′-flanking region bearing different haplotypes (F1Hs) found in the Han Chinese population. Promoter activity analysis revealed that the most common haplotype, F1H1 (namely A^−1684^/A^−1610^/C^−1196^/G^−462^), with an overall frequency of 92.1%, showed the highest transcriptional activity. The other two variant haplotypes, F1H2 (A^−1684^/T^−1610^/C^−1196^/G^−462^) generated less transcriptional activity than F1H1, while F1H3 (A^−1684^/A^−1610^/T^−1196^/A^−462^) exhibited the least activity of the three constructs. This result suggested that transcription may be more tightly regulated in F1H2 than in F1H3. Consistent with the result of core proximal promoter (601 bp, −540 nt to +61 nt) activity assay, the luciferase reporter gene assays also showed that haplotypes bearing the −462 G allele had higher promoter activity than those that carried the −462 A allele. Our results implied that different haplotypes composed of different genetic variants can influence the transcriptional activity of the *PPP2R2D*.

Bioinformatic analysis revealed that the −462 G>A variant is located in a recognition sequence for NF1, which implies that this portion of the 5′-flanking sequence may be important for NF1-mediated regulation of gene expression. The NF1 family is encoded by four genes, NF1/A, /B, /C, and /X, which express a large number of splice variants that can act either as transcriptional activators or repressors depending on cellular and promoter context [46], [47], [48], [49]. The proteins of NF1 family play a wide role in the replication of several vital DNA molecules and in the transcription of many cellular genes. A significant role for NF1 in regulating cellular growth, hormonal induction and repression, and oncogenic processes has been also described [46]. In this study, an EMSA revealed that the NF1 forms a complex with a DNA probe containing −462 G, but not with an identical probe in which G at position −462 nt was substituted for a −462 A allele. Supershift and cross-competition assay demonstrated that human NF1 binds to the proximal 5′-flanking region of *PPP2R2D* that contains −462 G variant. Introduction of exogenous NF1/B expression plasmid into L02 cells induced dose-dependent increase of the *PPP2R2D* promoter activity and also upregulated the expression of the endogenous *PPP2R2D*. Because NF1 can act as an activator or repressor of the same gene depending on the cell type or promoter context, it can be suggested that the variant −462 G>A with different affinity for NF1 might help to alter transcriptional activity and, thus, increase mRNA and protein expression of *PPP2R2D*. In accordance with this hypothesis, our results further confirmed that the NF1 may implicate in the positive control of PP2A-Bδ expression.

This study reveals, for the first time, the occurrence of variations and distinct haplotypes in the 5′-flanking region of *PPP2R2D* in the southern Han Chinese population. We provide data to indicate that the −462 G>A variant can functionally regulate the transcriptional activity of *PPP2R2D*. The data provide support for further investigation into the molecular and cellular mechanisms behind the significant allelic differences between haplotypes and the effect of different haplotypes on the regulation of *PPP2R2D*. In the future, our haplotype information should allow for more efficient association studies, permit comparisons with studies of other ethnicities and help to identify people who are susceptible to damage or disease linked to these genes.
